# Coagulopathy and its associated factors among patients with a bleeding diathesis at the University of Gondar Specialized Referral Hospital, Northwest Ethiopia

**DOI:** 10.1186/s12959-021-00287-6

**Published:** 2021-06-01

**Authors:** Melak Aynalem, Elias Shiferaw, Yemataw Gelaw, Bamlaku Enawgaw

**Affiliations:** Department of Hematology and Immunohematology, School of Medical Laboratory Sciences, College of Medicine and Health Sciences, University of Gondar, P.O. Box 196, Gondar, Ethiopia

**Keywords:** Bleeding diathesis, Coagulopathy, Gondar, Ethiopia, Mixing test

## Abstract

**Background:**

Coagulopathy is the major cause of mortality and morbidity throughout the world. Globally, about 26–45% of healthy people have a history of bleeding symptoms, which may be a result of thrombocytopenia, factor deficiency, or pathological inhibitory.

**Objective:**

To assess coagulopathy and its associated factors among patients with bleeding diathesis at the University of Gondar Specialized Referral Hospital from January to May 2020.

**Method:**

A cross-sectional study was conducted on 384 study participants with bleeding diathesis recruited by using a convenient sampling technique. Socio-demographic and clinical characteristics were collected by using questioners. Then 6 ml venous blood was collected with a needle and syringe method. About 3 ml blood was transferred to EDTA test tube for platelet count and 2.7 ml blood was transferred to a test tube containing 0.3 ml of 3.2% sodium citrated anticoagulant for coagulation test. For those study participants with prolonged coagulation tests, a mixing test was done. Blood film and stool examination were also done for malaria and intestinal parasite identification, respectively. The data were entered into EPI-Info version 3.5.3 and then transferred to SPSS version-20 for analysis. Descriptive statistics were summarized as percentages, means, and standard deviations. Bivariate and multivariate logistic regression was used to identify the associated factors, and a *P*-value less than 0.05 was considered statistically significant.

**Results:**

In this study, the prevalence of coagulopathy was 253/384 (65.9%; 95% CI: 61.16, 70.64). From them, 21.3% (54/253), 51.4% (130/253), and 27.3% (69/253) had only thrombocytopenia, only prolonged coagulation test, and mixed abnormality, respectively. Among participants with prolonged coagulation time, the prevalence of factor deficiency was 21.1% (42/199). Cardiac disease (AOR = 4.80; 95% CI: 2.65, 23.1), and other chronic diseases (AOR = 8.1; 95% CI: 1.84, 35.58) were significantly associated with coagulopathy.

**Conclusion:**

In this study, coagulopathy due to inhibitory was a public health problem. The participants with cardiac and other chronic diseases were at high risk for coagulopathy. Therefore, mixing tests could be done for all prolonged coagulation tests and it could be considered as a routine laboratory test.

## Background

Hemostasis is a process that prevents and stops bleeding, it is important to keep blood within a damaged blood vessel [1]. The normal hemostatic system comprises four parts namely vasculature, platelets, coagulation factors, and fibrinolytic proteins [2]. A defect in any of these compartments can result in coagulopathy [3].

Hemostasis can be classified as primary and secondary. Primary hemostasis is the first-line response to endothelial damage. When vascular endothelium is damaged, local vasoconstriction is initiated and platelets will be activated. This results in the formation of temporal platelet plug [4, 5], and initiates secondary hemostasis or coagulation. Secondary hemostasis involves the sequential activation of multiple coagulation factors a process that ultimately results in the formation of a stable fibrin clot over the already formed platelet plug. Finally, the formed blood clot will be removed by the fibrinolytic system [4].

Coagulopathy can be classified as primary or secondary. Primary hemostasis disorder includes a defect in blood vessels or platelets [5]. Whereas, secondary disorders involve qualitative or quantitative defects in clotting factors or their inhibitors [4]. Inhibitors can be anticoagulants, specific factors inhibitors, direct thrombin inhibitors, or non-specific inhibitors [6, 7]. On the other hand, acquired coagulation disorder is mainly associated with chronic diseases like liver disease, vitamin K deficiency, disseminated intravascular coagulation (DIC), and anticoagulant therapy [8, 9]. To assess these disorders laboratory tests like complete blood count (CBC), activated partial thromboplastin time (aPTT), prothrombin time (PT), and mixing test are ordered to investigate a defect in platelet number, factor deficiency, and presence of circulating coagulation factor inhibitors [6].

Coagulopathy is a major cause of public health problems, which results in morbidity or mortality worldwide [10]. About 26–45% of the world population who considered healthy had a history of nose and gum bleeding and about 5 - 10% of reproductive age women seek treatment for prolonged bleeding during the menstruation period [11].

Thrombocytopenia, vWD, and hemophilia are the leading causes of coagulopathy [10]. The worldwide incidence of vWD, hemophilia A, and Hemophilia B affects approximately 125 cases, 20.6 cases, and 5.3 cases per million populations respectively [12]. Studies showed that patients defect in vWD, platelet function, and coagulation factor was reported among 36.4–53%, 8.4–47%, and 3.9–23% of them, respectively [13]. Study among prolonged coagulation time participants 2.5% up to 77% were due to factor inhibitory [5, 14, 15]. Furthermore, coagulopathy can be also related to chronic diseases like liver disease, diabetic mellitus (DM), parasitic infection, and cardiovascular disease [16].

We proposed this study in Ethiopia particularly in the study area, due to many reasons. In Ethiopia, there is a high burden of malnutrition, infectious disease, and chronic disease. According to the global nutrition report, Ethiopia has a higher magnitude of stunting and wasting than the average for the African region (29.1%) [17]. Second, the magnitude of infectious diseases such as malaria, diarrhea, and intestinal helminthiasis, acute respiratory infections including pneumonia, tuberculosis, and skin diseases are still the country’s problem. Chronic diseases are also a higher burden to the country [18]. The above-mentioned diseases can cause coagulopathy. They can cause blood coagulation factors deficiency, production of blood coagulation factors inhibitors, and thrombocytopenia. In turn, these problems will lead to coagulopathy problems. Thus, this study was initiated to assess the magnitude of coagulopathy among patients with bleeding diathesis.

According to our knowledge, this study is the first study that can illustrate the coagulation factor deficiency and pathogenic blood coagulation factor inhibitory among patients with bleeding diathesis in Ethiopia particularly in the study are, Gondar. There was no previous study that had been conducted concerning evaluating the coagulation status of bleeding diathesis patients. Therefore, this study was intended to assess and fill the information gap of the current types of coagulopathy among bleeding diathesis patients which is not well elaborated in the current study area.

## Materials and methods

### Study setting and study population

A Hospital-based cross-sectional study design was used to determine coagulopathy and its associated factors among bleeding diathesis patients. This study was conducted at the University of Gondar Specialized Referral Hospital from January to May 2020. The Hospital is found in Gondar town which is 737 km away from Addis Ababa, the capital city of Ethiopia. The hospital is providing different medical services to more than 7 million people in the region and peoples of the neighboring region.

A total of 384 study participants aged 2–84 years were included. Study participants with one major bleeding symptom from epistaxis, bleeding gums, prolonged menstrual bleeding, or prolonged bleeding after surgery or blood draw, were included. Besides, patients with more than two symptoms of bruising, petechial, purpura, excessive bleeding after a dental procedure, prolonged bleeding during vaccinations, bleeding from the rectum, hematuria, blood in a stool, or blood in the vomit were included. But study participants taking anticoagulant therapy, antiplatelet drugs, participants who had a history of snakebite within one month, with active bleeding due to trauma, and critically ill and unable to give informed consent were excluded from the study.

### Operational definitions

Coagulopathy: - is an abnormality of one of the hemostasis compartments; thrombocytopenia, abnormal high PT/international normalized ratio (INR) or APTT).

Thrombocytopenia: - platelet count less than 150,000 × 10^3^ [9],

Abnormal high PT= > 16 Seconds, Abnormal high INR > 1.2 [19].

Abnormal high APTT= > 36 Seconds [19].

### Data collection procedures

#### Socio-demographic and clinical data collection

A pre-tested structured questionnaire was used to obtain socio-demographic characteristics of study subjects via face-to-face interviews. The questionnaire includes variables for the assessment of the socio-demographic characteristics mainly gender, age, residence, educational status, religion, marital status, and occupation**.**

Clinical data were also collected using a data collection sheet with physical examination and medical record review. Family history of bleeding, history of drug intake within two weeks, physical exercise, smoking habits, taking any traditional medicine was collected.

### Sample collection and laboratory analysis

#### Blood sample collection

After the study participant has given a written informed consent or assent form, a venous blood sample was collected by laboratory technologists by using a syringe and needle collection system. A total of 6 ml whole blood was collected and then 3 ml blood was transferred to ethylene di-amine tetra acetic acid (EDTA) test tube for CBC analysis and 2.7 ml blood was transferred to 3.2% sodium citrate anticoagulated test tube for coagulation test. Blood was collected considering 1 sodium citrate to 9 blood and 1.5 mg EDTA to 1 ml blood proportion [20] for coagulation and CBC analysis, respectively. The quality of the sample was maintained by samples checking whether they were in the acceptable criteria like; hemolysis, clotting, volume, and collection time.

#### Platelet count

The platelet count was done by Sysmex KX-21 hematology analyzer. Sysmex KX-21 is an automatic multi-parameter (18 parameters) blood cell counter for in vitro diagnostic use in clinical laboratories. The counting is based on the impedance principle in which a constant electric current is passed through a solution. Then it measures the changes in electrical resistance that occur when blood cells pass through the detection aperture [21]. For the CBC analyzer, data quality control and analyzer maintenance were done by using Clinical Laboratory Institute for Standardization standard (CLIS) [20].

#### Coagulation tests (PT/INR, and APTT)

Coagulation profile tests (PT/INR, and aPTT) were analyzed by Huma cue-due plus (Human diagnostic Worldwide, Germany) semi-automated analyzer which uses the turbidity meter principle. For the coagulation test, the quality control was done by using normal and abnormal lyophilized samples daily before the patient sample was run. The maintenance for machines was done as CLIS standard [20]. On occasions where there was a delay, the sample was stored at room temperature, and 4^o^c [20].

Prothrombin time and Activated Partial thromboplastin time were analyzed on an automated instrument at 37 °C which acts like a normal human body temperature. A blood sample was drawn into a test tube that containing liquid sodium citrate. Platelet poor plasma (PPP) was prepared from the blood sample by centrifugation. Then, platelet poor plasma was incubated at 37^o^c, and mixed with thromboplastin reagent. The time taken from the addition of thromboplastin reagent to the formation of the fibrin clot was measured by the automated system as the PT/INR. But, to do aPTT reagent one an excess of Cacl_2_ was mixed with PPP and incubated for 3 min. Finally, to activate the intrinsic pathway of coagulation, reagent two (Kaolin) was added to the mixture of PPP and reagent one as an activator. Then time from the addition of reagent II to clot was measured optically [20].

#### Preparation of Normal pooled plasma

Pooled normal plasma which was used for mixing test contains citrated plasma and it was prepared from 30 carefully screened normal human donors. An approximately equal number of male and female donors were included. First, PPP was prepared for all donors and it was analyzed for PT/INR, and aPTT tests. Second, only donors with normal results of PT/INR, and aPTT tests were mixed in a single test tube called normal pooled plasma (NPP). Finally, the above test was repeated. If the NPP had a normal result it was stored in the deep freezer. This NPP was used for mixing studies in the determination of a prolonged PT and/or APTT [22].

#### Mixing test

Mixing studies were tests performed on citrated PPP, which were used to distinguish factor deficiencies from factor inhibitors. Inhibitors can be lupus anticoagulants, or specific factor inhibitors, such as antibodies directed against factor VIII. Mixing study works as the fact, factor levels up to 50% lower than the normal value can give a normal PT or APTT. The Principle was based on the fact that patient plasma is mixed 1:1 with NPP that contains 100% of the normal factor level results in a level of ≥50% in the mixture. If the abnormal result was corrected by the addition of NPP, a factor deficiency is indicated. Whereas, when there was no correction of the abnormal result, it indicates the presence of a circulating inhibitor [22].

#### Immediate mixing test

First 1:1 dilution of patient PPP using NPP as the diluent was prepared. Then an equal volume of PPP was mixed with NPP. Then, we had mixed the plasma carefully. Finally, we had measured immediately the PT/INR, and APTT for the immediate mixing test. The interpretation was done by the following rules. If the APTT or PT was corrected by NPP at the immediate phase, a factor deficiency or weak inhibitors were indicated. If the APTT or PT were not corrected by the addition of NPP immediately, a strong inhibitor is indicated [22].

#### Incubated mixing test

First 1:1 dilution of patient PPP using NPP as the diluent was prepared. Then we had carefully mixed the participant PPP with NPP and the solution was incubated at 37^o^c for 1 up to 2 h. Finally, we had measured the APTT or PT test. The interpretation was done by the following rules. If the APTT or PT was corrected by NPP at the incubation phase, a factor deficiency was indicated. If the APTT or PT is not corrected by the addition of NPP, weak (mainly IgG antibody) inhibitors were indicated [22].

#### Stool examination

To determine the association of coagulopathy and intestinal parasite a pea-sized stool was collected by labeled, clean, leak-proof, wide mouse containers. Then wet mount was prepared by using normal saline and direct microscopy was done by using a light microscope. We used formal ether sedimentation technique for the concentration of stool parasites [23].

#### Blood film examination for malaria parasites

Malaria was diagnosed by using light microscopic examination, stained with 10% Giemsa. Peripheral blood smear (thick and thin blood film) was prepared by collecting blood and smeared on a clean microscope slide. The slide allowed to air dry and then fixed with methanol, stained, and examined by laboratory technologists.

### Data quality control

#### Sociodemographic and clinical data quality control method

The questionnaire was prepared in English and translated to Amharic (local language) then translated back to English to check for consistency. All study participants were informed about the aim and importance of the study before data collection to make them fully concerned about their response. The questionnaire was pre-tested and training was given for data collectors. The collected data were checked daily for consistency and accuracy. Data collection was closely supervised by investigators. To avoid hemolysis blood collection and blood handling were conducted by following all protocols.

### Statistical analysis

Data were entered using EPI-Info version-3.5.3 then transported to SPSS version 20 for analysis. Skewness and kurtosis were used to check data distribution and the data were normally distributed. Then, descriptive statistics were summarized as percentages, means, and standard deviations and presented with figures and tables. Each of the outcome variables was computed with each independent variable. The association of the independent variable with the categorical outcome variable was measured by calculating the odds ratio with a 95% confidence interval using bivariate and multivariate logistic regression analysis. Variables having a *p*-value of less than 0.2 were selected for multivariate logistic regression analysis. *P*-value < 0.05 was considered to be statistically significant.

## Results

### Sociodemographic characteristics

The current study included a total of 384 study participants; 210 (54.7%) of them were male, and 249 (64.8%) from an urban residence. The mean age of the study participants was 37 ± 19 years ranging from 2 to 87 years. The majority of 234 (60.9%) and 257 (66.9%) of the study participants were in the age range of 18–45 years and married respectively (Table 1).

**Table 1:** Sociodemographic characteristics of the study participants

Variables	Categories	Frequency	Percentages
Sex	Male	210	54.7%
	Female	174	45.3%
Age in years	< 18	39	10.2%
	18–45	234	60.9%
	46–65	69	18.0%
	> 65	42	10.9%
Residence	Urban	249	64.8%
	Rural	135	35.2%
Educational level	Unable to read and write	56	14.6%
	Attend primary school	106	27.6%
	Attend secondary school	42	10.9%
	Attend higher education	180	46.9%
Religion	Orthodox	279	72.7%
	Muslim	83	21.6%
	Other	22	5.73%
Occupational status	Employed	79	20.6%
	Student	91	23.7%
	House wife	83	21.6%
	Farmer	66	17.2%
	Other	65	16.9%
Marital state	Married	257	66.9%
	Un married	127	33.1%

### Characteristics of clinical characteristics

About 212 (55.2%) of the study participants had a history of chronic disease. On the other hand, 207 (53.9%) of them were taking different medications. Of the total study participants, 36 (11.6%) of them were anemic. Of the total of 384 study participants, 71 of them had intestinal parasites and 21 of the study participants had malaria. Study participants taking a different type of medication and liver diseased individuals were also had higher thrombocytopenia, and coagulation disorder (Tables 2 and 3).

**Table 2:** Clinical characteristics of study participants

Characteristics		Frequency	Percentages
**Pregnancy (Female** ***n*** **= 174)**	**Yes**	18	4.70%
**Family History with Bleeding**	**Yes**	35	9.10%
**Hypertension**	**Yes**	36	9.40%
**Cardiac Disease**	**Yes**	84	21.90%
**Other Chronic Disease**	**Yes**	128	33.30%
**Drug Taking**	**Yes**	207	53.90%
**High Protein and Vitamin Food Intake**	**Yes**	309	80.50%
**Physical Exercise**	**Yes**	131	64.10%
**Smoking**	**Yes**	10	2.60%
**Alcohol Drinking**	**Yes**	40	10.40%
**Traditional Medicine (within 2 weeks)**	**Yes**	20	5.20%
**Malaria Infection**	**Yes**	21	5.50%
**Intestinal Parasite Infection**	**Yes**	71	18.50%

**Table 3:** Characteristics of Coagulopathy abnormality

Study subject characteristics		Coagulopathy abnormalities							
		Thrombocytopenia		Prolonged PT		Prolonged APTT		Factor deficiency	
		Yes	No	Yes	No	Yes	No	Yes	No
Gender	Male	82	128	108	102	12	198	32	178
	Female	41	133	91	83	19	155	10	164
Age in year	< 18	13	26	8	31	1	38	3	36
	18–45	76	158	122	112	18	216	27	207
	46–65	25	44	41	28	6	63	8	61
	> 65	9	33	28	14	6	36	4	38
Residence	Urban	75	174	121	128	17	232	22	227
	Rural	48	87	78	57	14	121	20	115
Educational level	None	58	122	101	79	14	166	20	160
	Primary school	35	71	54	52	6	100	13	93
	Secondary school	14	28	16	26	5	37	4	38
	Higher education	16	40	28	28	6	50	5	51
Bleeding History	Yes	14	21	20	15	6	29	2	33
	No	109	240	179	170	25	324	40	309
Hypertension	Yes	9	27	17	19	3	33	2	34
	No	114	234	182	166	28	320	40	308
Cardiac disease	Yes	10	74	74	10	16	68	2	82
	No	113	187	125	175	15	285	40	260
Drug intake	Yes	77	130	133	74	24	183	20	187
	No	46	131	66	111	7	170	22	155
Physical exercise	Yes	46	85	58	73	9	122	19	112
	No	77	176	141	112	22	231	23	230
Smoking habit	Yes	6	4	8	2	1	9	2	8
	No	117	257	191	183	30	344	40	334
Alcohol consumption	Yes	42	44	56	30	6	80	12	74
	No	81	217	143	155	25	273	30	268
Traditional medicine	Yes	18	22	28	12	2	38	9	31
	No	105	239	171	173	29	315	33	311
Malaria infection	Yes	6	15	20	1	1	20	14	7
	No	117	246	179	184	30	333	28	335
Intestinal Parasite	Yes	18	53	32	39	5	66	5	66
	No	105	208	167	146	26	287	37	276

### Laboratory findings

**Fig. 1:**
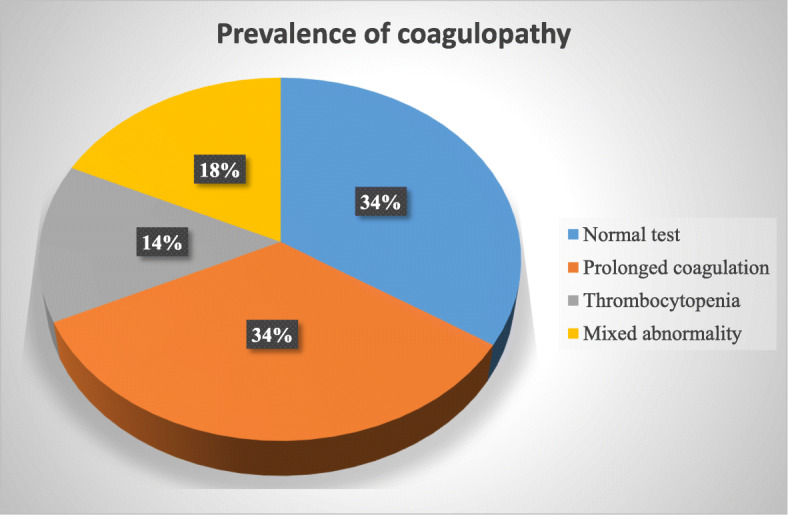
Prevalence of coagulopathy among study participants

**Fig. 2:**
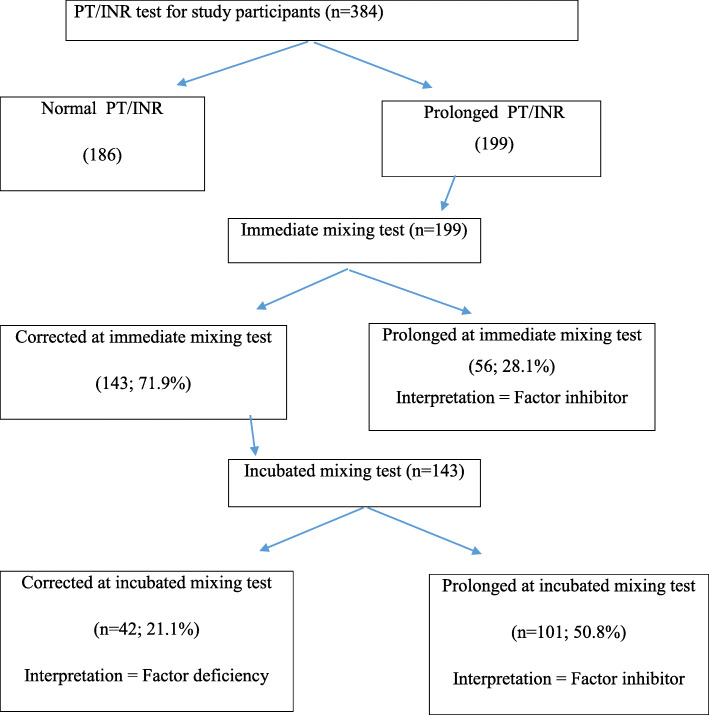
Mixing test for prolonged PT test among study participants

**Fig. 3:**
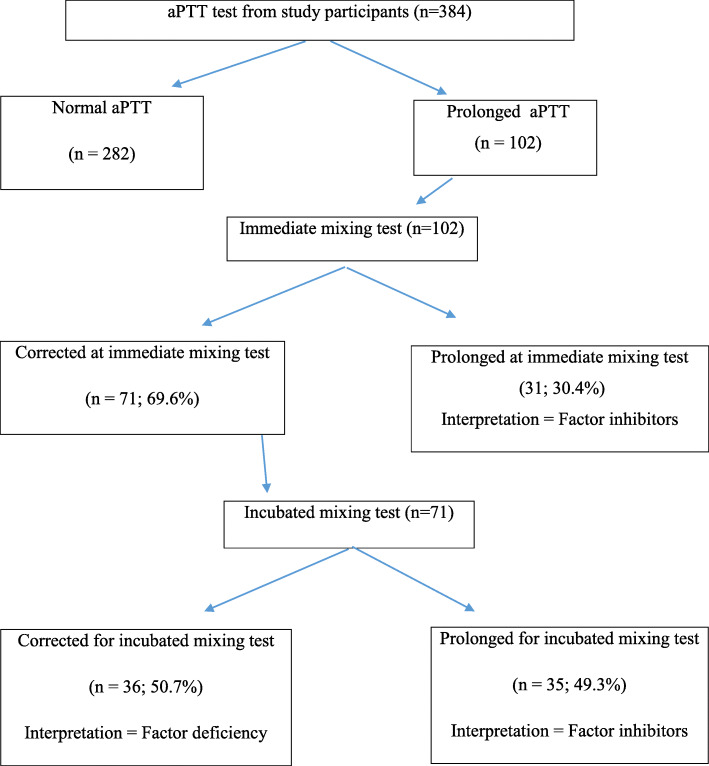
Mixing test for prolonged aPPT test among study participants

The overall prevalence of coagulopathy was 253 (65.9%; 95% CI: 61.16, 70.64). From the total study participants, 199 (51.8%) showed prolonged coagulation time (prolonged PT and aPTT) and 123 (32%) thrombocytopenia. Of the 199 study participants with prolonged coagulation time, 21.1% (42/199) and 78.9% (157/199) were due to the presence of factor deficiency and factors inhibitors, respectively (Fig. 1). Furthermore, the prevalence of prolonged PT/INR test was 51.8% (199/384). From them, the prevalence of factor deficiency and inhibitors were 21.1% (42/199) and 78.9% (157/199), respectively (Fig. 2). Also, the prevalence of prolonged aPTT test was 26.6% (102/384). From this, the prevalence of factor deficiency and inhibitors were 35.3% (36/102) and 64.7% (66/102) respectively (Fig. 3).

### Factors associated with coagulopathy

In bivariate logistic regression analysis study participants with cardiac disease (COR = 6.60; 95% CI: 3.07, 14.17), other chronic diseases (nasal bleeding, anemia, diabetic mellitus, liver disease) (COR = 4.83; 95% CI: 2.77, 4.83), drug intake (COR = 4.42; 95% CI: 2.81, 6.95), and alcohol intake (COR = 2.02; 95% CI: 1.23, 3.33) showed association with coagulopathy. Therefore, these variables and other variables with a *p*-value of less than 0.2 were subjected to multivariable binary logistic regression. However, in multivariable analysis, cardiac disease (AOR = 10.76; 95% CI: 4.2, 27.58), and other chronic diseases (AOR = 6.9; 95% CI: 3.52, 13.55) were significantly associated with coagulopathy (Tables 4 and 5).

**Table 4:** Factors associated with coagulopathy

Study Participant characteristics		Coagulopathy		COR (95% CI)	***P*** Value	AOR (95% CI)
		Yes N (%)	No N (%)			
Gender	Male	139 (66.2%)	71 (33.8%)	1	0.89	–
	Female	114 (65.5%)	44 (34.5%)	1.03 (0.67, 1.57)		–
Age in years	< 18	18 (46.2%)	21 (53.8%)	2.05 (1.03, 4.05))	0.001	2.97 (0.74, 11.99)
	18–45	149 (63.7%)	85 (36.3%)	1		1
	46–65	54 (78.3%)	15 (21.7%)	0.49 (0.26,0.92)		2.72 (0.92, 8.09)
	> 65	32 (76.2%)	10 (23.8)	0.55 (0.26, 1.15)		2.04 (0.66, 6.29)
Residence	Urban	204 (81.9%)	45 (18.1%)	0.88 (0.50–1.54)	0.113	1.12 (0.61, 2.05)
	Rural	113 (83.7%)	22 (16.3%)	1		1
Educational level	None	129 (56.1%)	51 (43.9%)	0.123 (0.327, 0.611)	0.115	0.98 (0.38, 2.51)
	Primary school	67 (50.9%)	39 (49.1%)	0.755 (0.462, 0.900)		1.22 (0.63, 2.37)
	Secondary school	23 (38.1%)	19 (61.9%)	0.555 (1.27, 0.568)		0.62 (0.25, 1.53)
	Higher education	34 (50%)	22 (50%)	1		1
Occupation	Employed	46 (58.2%)	33 (41.8%)	1		1
	Student	45 (49.5%)	46 (50.1%)	1.45 (0.58, 3.62)	0.001	0.68 (0.27, 1.70)
	House wife	63 (75.9%)	20 (24.1%)	0.85 (0.34, 2.13)		1.18 (0.47, 2.93)
	Farmer	53 (80.3%)	13 (19.7%)	0.34 (0.16, 0.73)		2.97 (0.98, 7.86)
	Other	46 (70.8%)	19 (29.2%)	0.58 (0.29, 1.56)		1.63 (0.66, 4.07)
Marital status	Never married	74 (45.7%)	53 (54.3%)	1.64 (1.06, 2.56)	0.27	–
	Married	179 (69.6%)	78 (30.4%)	1		–

**Table 5:** Factors associated with coagulopathy

Study Participant	Coagulopathy			COR (95% CI)	***P*** Value	AOR (95% CI)
	Yes		No			
Family Bleeding History	Yes	28 (80%)	7 (20%)	2.20 (0.84, 5.19)	0.071	2.54 (0.94, 6.88)
	No	225 (35.5%)	124 (64.5%)	1		1
Pregnancy	Yes	9 (50%)	9 (50%)	0.50 (0.19, 1.29)	0.152	0.65 (0.19, 2.13)
	No	122 (33.3%)	244 (66.7%)	1		1
Hypertension	Yes	23 (63.9%)	13 (36.1%)	0.91 (0.44, 1.85)	< 0.001	–
	No	230 (66.1%)	118 (33.9)	1		–
Cardiac Disease	Yes	76 (90.5%)	8 (9.5%)	6.60 (3.07, 14.17)	0.791	10.76 (4.2, 27.58) *
	No	177 (59%)	123 (41%)	1		1
Other chronic disease	Yes	110 (75%)	18 (25%)	4.83 (2.77,4.83)	< 0.001	6.9 (3.52, 13.55) *
	No	143 (34.4%)	113 (65.6%)	1		1
Drug taking	Yes	167 (80.7%)	40 (19.3%)	4.417 (2.81, 6.95)	< 0.001	1.731 (0.89, 3.35)
	No	86 (48.6%)	131 (51.4%)	1		1
Physical exercise	Yes	82 (62.6%)	49 (37.4%)	1	0.328	–
	No	171 (67.6%)	82 (32.4%)	1.25 (0.52, 1.25)		–
Alcohol consumption habit	Yes	56 (65.1%)	30 (34.9%)	2.02 (1.23, 3.33)	0.060	1.84 (0.95, 3.58)
	No	143 (47.9%)	155 (52.1%)	1		1
Traditional medicine	Yes	28 (70%)	12 (30%)	2.36 (1.16, 4.79)	0.202	–
	No	171 (49.7)	173 (50.3%)	1		–
Intestinal parasite infection	Yes	32 (45.1%)	39 (54.9%)	0.72 (0.43, 1.20)	0.007	0.50 (0.26, 1.15)
	No	167 (53.3%)	146 (46.7%)			1

## Discussion

Coagulopathy is a global public health problem, which results in mortality and morbidity [10]. Thrombocytopenia, vWD, and hemophilia are the leading causes of coagulopathy [10]. The effect of bleeding disorder in Africa and Ethiopia is considered a public health problem [11, 24]. Hence, this study was aimed to assess the prevalence and associated risk factors of coagulopathy among bleeding diathesis participants attending at University of Gondar Specialized Referral Hospital.

The finding of this study showed that the overall prevalence of coagulopathy was (65.9%; 95% CI: 61.16, 70.64). This result is considered a high public health problem. The reason may be associated with the type of study participants included in this study. More than half (55.2%) of them were with different types of chronic diseases. From this liver disease, cardiac disease, and diabetes Miletus (DM) was the majority of them. These diseases are directly associated with coagulopathy [12, 25–29]. Coagulopathy due to liver disease is a result of all coagulation factors involved in the generation of a fibrin clot, and thrombopoietin is produced by liver cells [30]. Also, coagulopathy due to DM is mostly related to thrombocytopenia. In contrast, glycation of hemoglobin, prothrombin, fibrinogen, and other proteins involved in the clotting mechanism results in a hypercoagulable state [29]. On the other hand, coagulopathy due to cardia disease is mostly related to medications that are given to the patients. The drugs that are associated with thrombocytopenia and prolonged coagulation test include glycoprotein IIb/IIIa receptor inhibitors, heparin, warfarin, and thienopyridines [31].

The current study was in agreement with a study conducted by Tapia et al. in American which reported a 65.6% prevalence of bleeding disorder [13]. In contrast, the prevalence of this study was higher than a study conducted in Egypt (23%) [5], India (53%) [32], and America (47%) [14]. The possible reasons for the discrepancies might be associated with differences in the study population, study period, geographical variability, detection method, and implementation of different strategies to minimize the burden of coagulopathy in the region where studies have been conducted.

In this study, the prevalence of thrombocytopenia was (32%; 95% CI: 27.3, 36.7) which was nearly one out of three participants was with thrombocytopenia. Thrombocytopenia was commonly associated with bleeding diathesis patients. It can be caused by malnourishment, liver disease, bone marrow disease, sepsis, DIC, heparin, certain antibiotics, and different chronic diseases [29, 33]. Most of the above causes were detected in the current study participants. This may be the reason for the moderate result of thrombocytopenia. The current study finding was higher than a study conducted by David et al. in Canada which showed a 13.3% prevalence of thrombocytopenia [25]. In contrast, this study finding was lower than the study conducted in America (47.6%) [34] and India (38%) [33]. The variability may be related to differences in study population and variability in socio-economy.

In the current study, the prevalence of coagulopathy due to factor inhibitory and factor deficiency among prolonged coagulation tests were 78.9% (157/199), and 21.1% (42/199) respectively. This study indicated that four out of five individuals with prolonged coagulation tests were due to factor inhibitors. This might be related to the presence of chemicals, lupus anticoagulants, and specific immunoglobulin. Study participants in the current study were having different types of chronic diseases which may be the cause for the presence of high factor inhibitory. The prevalence of factor inhibitory (78.9%; 95% CI: 74.82, 82.89) was consistent with a study conducted by Kershaw et al. in Australia 77% [22]. On the other hand, the current study finding was higher compared to the study conducted in France (69%) [35], Italy (2.5%) [36], and Israel (67%) [37]. These studies showed that a higher level of factor inhibitors was detected than the factor deficiency. The variety of the result may be related to the type of study population used, study design, sample size, and all the above study were conducted on single factor deficiency type but this study was conducted on the presence of all type of factor deficiency as well as this study assessed presence of all type of inhibitory.

Coagulopathy is mostly associated with chronic diseases [25, 31, 38, 39], parasitic infection [40], and some viral agents [32]. In this study cardiac disease, and other chronic diseases were significantly associated with coagulopathy. Study participants with cardiac disease were nearly eleven times more likely to develop coagulopathy than those without cardiac disease. This might be due to cardiac study participants might take different types of medication which might have an impact on the normal hemostasis process [28]. Researchers conducted on the association of vWD factor deficiency with cardiovascular disease and asymptomatic carotid atherosclerosis By Seaman et al. in America showed that the prevalence of the cardiovascular disease among VWF deficient participants was 5.8% [16]. Similarly, research conducted in America by Mohamed et al. among cardiac patients showed that cardiac disease is associated with thrombocytopenia in which 10.17% of the cardiac patients had thrombocytopenia [28]. Both the above studies and the current study indicates that cardiac disease patients are a risk for prolonged coagulation test and thrombocytopenia.

In this study, other chronic diseases (nasal bleeding, anemia, DM, and liver disease) were statistically associated with coagulopathy. Those study participants who had other chronic disease were almost 7 times more likely to be coagulopathy than who had no other chronic diseases. The liver disease had an association with coagulopathy because all coagulation factors and thrombopoietin are produced in the liver cells. Also, a study shows all 3 phases of hemostasis were reduced among liver disease patients [27]. patients with liver disease had decreased synthesis of Vitamin K-dependent and independent clotting factors, reduced production of anticoagulants, platelet production abnormalities, and platelet consumption are the leading cause for prolonged coagulation test, and thrombocytopenia [41]. Similarly, DM patients are a risk for thrombocytopenia. Thrombocytopenia due to DM patients is commonly related to medications given to patients like insulin and autoimmunity to bone marrow cells. Shortened coagulation tests were also detected in DM participants. Research conducted by Richard et al. among Type-2 DM in America and with other previously published reports showed that shortened APTT and PT in diabetes patients compared to non-diabetic controls [29]. Also, research conducted by Acang et al. in Indonesia shows that type 2 DM study participants were exposed to hypercoagulability [38]. Similarly, research conducted by Erem et al. in Kuwait shows, the plasma levels of fibrinogen, antithrombin III, plasminogen activator inhibitor-1, VWF activity, and PT were found to be significantly increased in the type 2 DM patients compared with the healthy subjects [26].

The first major limitation of this study was being cross-sectional nature that does not allow us to observe causality in the relationship. Due to the constraint of resource, we did not perform advanced techniques which can assess specific factors and concentration technique to assess a small number of parasites. Moreover, recall bias might be a possible factor in the assessment of past events and exposures.

## Conclusion

The present study demonstrated that the prevalence of coagulopathy among bleeding diathesis at the University of Gondar Specialized Referral Hospital was a high public health problem. Participants with bleeding diathesis can experience multiple bleeding disorders. Prolonged APTT, PT, and thrombocytopenia were found among study participants with bleeding diathesis. As shown by this study, factor inhibitors were more prevalent than other causes of coagulopathy. Also, cardiac disease and other chronic diseases were the contributing factors for the development of coagulopathy among study participants.

The governmental body and health workers could understand the distribution of factor inhibitory effect on bleeding diathesis patients and mixing tests must be considered as a routine laboratory test and should be applied for every prolonged coagulation result. Furthermore, the researcher’s further studies on longitudinal study design could be conducted to identify the cause-effect relationships with its contributing factors. Studies should be conducted by including the specific factor test and genomic test for mutation of a gene to assess hemophilia type. Not only specific factors but also inhibitory types that are commonly found should be clarified.

## Data Availability

All data supporting these findings is contained within the manuscript.

## Abbreviations

aPTT: Activated Partial Thromboplastin Time
CBC: Complete Blood Count
DIC: Disseminated Intravascular Coagulation
DM: Diabetic Mellitus
EDTA: Ethylene Di-amine Tetra Acetic acid
INR: International Normal Ratio
NPP: Normal Pooled Plasma
PPP: Platelet Poor Plasma
PT: Prothrombin Time
vWD: von Will brand Disease
vWF: von Will brand Factor
